#  Gold and Iron Oxide Nanoparticle-Based Ethylcellulose Nanocapsules for Cisplatin Drug Delivery

**Published:** 2011

**Authors:** Kannaiyan Sathish Kumar, Vasudevan Jaikumar

**Affiliations:** *Department of Chemical Engineering, SSN College of Engineering, Kalavakkam 603 110, India.*

**Keywords:** Anticancer drug, Nanocapsules, Phosphate buffer, Drug release, Standard deviation, Coefficient of variation

## Abstract

The present study is aimed at the overall improvement in the efficacy, reduced toxicity and enhancement of therapeutic index of cisplatin. Nanocapsules of cisplatin containing ethylcellulose have been prepared using solvent evaporation technique under ambient conditions. The prepared nanocapsules were used for controlled drug release of anticancer agents with gold and iron oxide nanoparticles. The drug-entrapped nanocapsules were characterized by scanning electron microscopy (SEM) and transmission electron microscopy (TEM). Fourier transform infrared (FTIR) studies indicated the absence of chemical interactions between the drug, polymer and metal nanoparticles. The drug loaded nanoparticles are spherical in shape and had average diameter in the range of 100-300 nm. Drug release study showed that the acidic media provided a faster release than the phosphate buffer media. These findings were also compared statistically through calculating mean, standard deviation and coefficient of variation for various polymer nanocapsules. However, the drug release for gold nanoparticles/anticancer drug (Au-cis) incorporated ethylcellulose nanocapsules was controlled and slow compared to iron oxide nanoparticles-cisplatin incorporated ethylcellulose nanocapsules. Hence, gold nanoparticles act as good trapping agents which slow down the rate of drug release from nanocapsules.

## Introduction

Controlled drug delivery technology represents one of the frontier areas of science. These delivery systems offer numerous advantages compared to conventional dosage forms like improved efficacy, reduced toxicity and improved patient compliance and convenience. Research and development in the field of drug delivery systems facilitating site-specific therapy has achieved significant progression. Safe and non-toxic formulation of cytotoxic drug and its site specific delivery at its target-tumor tissue or tumor cells have become the major goal of the research. Such systems often use biodegradable polymers as drug carriers. (1). 

Ethylcellulose (EC), a biodegradable polymer, is one of the most useful polymers for drug delivery applications (2). Ethylcellulose is among a very small number of water-insoluble polymers that are approved for global pharmaceutical applications and is most frequently used in extended release solid dosage formulations. It can also be used for extended release multi-particulate coating, micro-encapsulation of actives, taste-masking of bitter actives, solvent and extrusion granulation, tablet binding for dry and direct compression and has shown good solubility in organic solvents. In addition to being useful in a variety of pharmaceutical applications, Ethylcellulose also provides formulation flexibility by accommodating a range of molecular weights and can be blended for intermediate viscosities. It also features a fine particle range for use in extended release matrix systems and provides improved lipophilic properties realized by the increased surface area. This flexibility is further enhanced by the ability to modify the release profiles when ethylcellulose is used in combination with the water-soluble excipients.

The preparation methods for nanoparticles depend on the nature (hydrophobic or hydrophilic) of the drug to encapsulate. There are different methods for the preparation of polymeric nanoparticles, among which, the emulsification solvent evaporation technique (3) is the most useful due to being simple, fast and economic and also having the advantage of employing non-toxic solvents.

**Figure 1:**
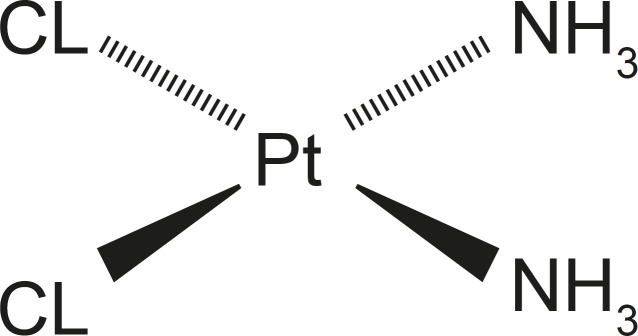
Structure of cisplatin

Cisplatin, an anticancer drug, is one of the most available, potent and effective anticancer drug these days however, its applications have been limited due to its serious side effects (4, 5). Cisplatin, cisplatinum or cis-diamminedichloridoplatinum (II) (CDDP) is a platinum-based chemotherapy drug (Figure 1) used to treat various types of cancers like small cell lung cancer, urinary bladder cancer and ovarian cancer and germ cell tumors. Platinum complexes are formed in cells, which bind and cause cross-linking of DNA ultimately triggering apoptosis, or programmed cell death. Cisplatin is administered intravenously as short-term infusion in physiological saline for treatment of solid malignancies (6).

Targeted delivery of cisplatin to the specific cancer cells is thus required as it can provide a means of modifying the distribution of cisplatin *in-vivo *and increasing its concentration in the target sites and thereby, improving efficacy and reducing toxicity. Several strategies have been described in the literature for selective cisplatin drug delivery. Some of these attempts include the administration of cisplatin by its water-soluble conjugates, such as with poly (amidoamines) (7, 8) and poly (amidoamines) dendrimers (9). The anticancer activity of cisplatin entrapped PLGA-methoxy poly (ethylene glycol) nanoparticles on prostate cancer cells and the *in-vitro *nanoparticle degradation, *in-vitro *drug release and *in-vivo *drug residence in blood properties of PLGA-methoxy poly (ethylene glycol) nanoparticles of cisplatin have also been investigated (10).

Although gold nanoparticles and iron oxide nanoparticles have attracted considerable attention due to their potential applications in molecular recognition systems and in drug delivery, less data were available utilizing metal nanoparticles to extend the controlled release time. Therefore, the objective of this study was to characterize the cisplatin-containing EC nanocapsules and determine quantitatively the effect of metal nanoparticles on the average size, the morphology and the cisplatin release.

## Experimental

Cisplatin was purchased from Dabur Pharmaceuticals Ltd. (Dabur, India). Ethylcellulose and all other solvents used in these studies were purchased from SRL India Ltd. 

*Apparatus*

Sample was characterized by ultraviolet-visible (UV-Vis) spectrophotometry (Perkin-Elmer Lambda 25). The path length was 1 cm and the matched 1 × 1 cm cuvettes were used. Transmission electron microscope (HR-TEM) was undertaken employing a JEOL instrument with an accelerating voltage of 120 KV. Sample was prepared by the drop-casting of a drop of nanogold suspension onto a 200 mesh copper grid and the subsequent air-drying. The morphology and surface characteristic of the microcapsules were examined with a scanning electron microscope (SEM, JEOL JSM-5410). Samples were first mounted onto a copper plate with a diameter of approximately 3 cm. These mounted samples were then dried in a vacuum oven at 40°C for 24 h. Fourier transform infrared spectroscopy (FT-IR) was performed using a PE IR SPECTRUM ASCII PEDS 1.60 spectrometer and samples were presented as KBr pellets. Spectra were acquired at room temperature at resolution of 4 cm^-1^.

*Preparation of citrate-capped gold nanoparticles*

**Figure 2:**
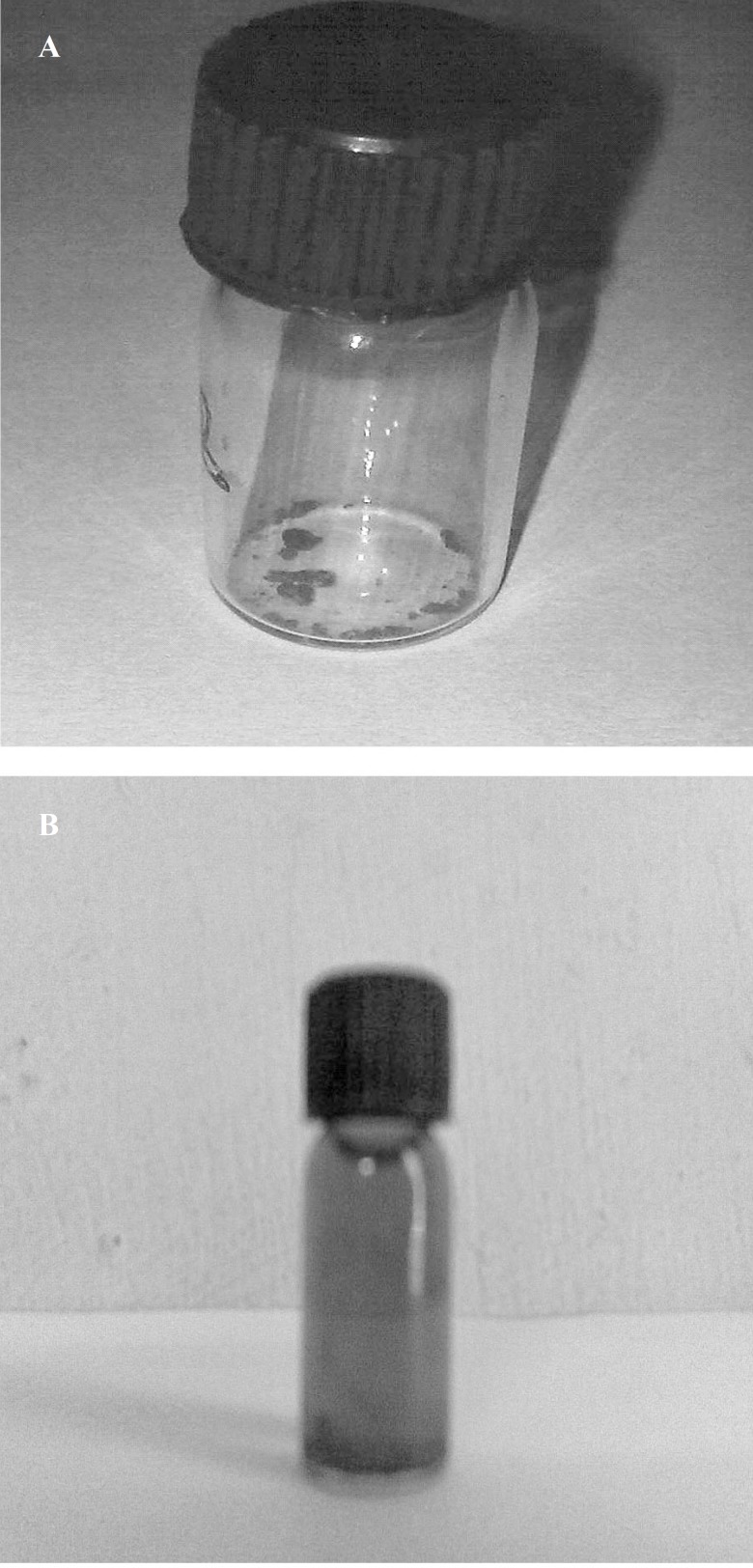
(a) Colloidal nanogold solution; (b) Iron oxide nanoparticles

A mass of 5.0×10^−6^ M Tetrachloroauric acid (HAuCl_4_) was taken dissolved in 19 mL of deionized water resulting in a faint yellowish solution. The solution was heated and 1 mL of 0.5% sodium citrate solution was added to the boiling solution and was stirred for the next 30 min. The faint yellowish color of the solution gradually changed to clear, grey, purple and deep purple, respectively, until settling on wine-red (Figure 2a). Water was added to the solution to bring the volume back up to 20 mL to account for evaporation.

*Preparation of oleic acid coated iron oxide nanoparticles*

A mass of 0.42 g of ferrous chloride (FeCl_2_) and 1.09 g Ferric chloride (FeCl_3_) were dissolved in 10 mL of 1 N HCl (11, 12) by mechanical stirring. This acid solution was added drop-wise to an aqueous solution (90 mL) of 1 N KOH under Nitrogen atmosphere leading to a black precipitate. The precipitate was isolated by decantation and washed several times with deionized water until the pH of the medium came to about 9. After the washing step, 20 mL of oleic acid was added to the alkaline medium containing magnetite nanoparticles under vigorous stirring for 1 h at room temperature. The wet precipitate was dried in an oven at 40°C for 48-72 h before being used. The resulting iron oxide nanoparticles are shown in Figure 2b.

Fe^2+^ + 2 Fe^3+^ + 8 OH− → Fe_3_O_4_ + 4H_2_O

*Preparation of ethylcellulose/cisplatin-gold nanocapsules (EC/Cis-Au)*

For EC/Cis-Au experiment, Cisplatin drug (50 mg) was added in 0.5 mL nanogold aqueous suspension (13) and this solution was added to an organic polymer solution (300 mg EC + 5 mL CH_2_Cl_2_) under stirring condition. One percent polyvinyl alcohol was also added as an emulsifier. This was continued until the organic solvent was completely evaporated. The suspension became clear after all the nanocapsules precipitated out of the solution. These nanocapsules were collected by filtration and washed with deionized water to remove any undesirable residuals. 

Finally, the clean nanocapsules were dried in a vacuum oven at 40°C for 24 h to ensure a complete removal of the organic solvent and deionized water. All the nanocapsules were stored in desiccators at 25°C. EC/Cis nanocapsules were also prepared under similar conditions.

*Preparation of ethylcellulose/cisplatin iron oxide nanocapsules (EC/cis-Fe*_3_ *O*_4_ *)*

The Fe_3_O_4_-containing polymeric nanoparticles were prepared by the solvent evaporation technique in oil/water emulsion with EC as the encapsulation material. One hundred mg of EC, 20 mg of Fe_3_O_4_ and 7.5 mg of Cisplatin were dissolved in 8 mL of dichloromethane and vortexed for 10 min to make the organic phase. The organic phase was then poured into 50 mL of stirred aqueous solution containing 1% polyvinyl alcohol as emulsifier. The formed o/w emulsion was then stirred at room temperature overnight with a magnetic stirrer to evaporate the organic solvent. The particles were collected by centrifugation at 10,000 rpm for 10 min and washed three times with deionized water. The nanoparticles were re suspended with 10 mL water and freeze-dried (Edwards freeze dryer, ESM 1342) for 2 days. The EC-Cis Nanoparticles without Au and Fe_3_O_4_ were prepared in the same manner. 

*In-vitro release studies*

At first, a precise known amount of nanocapsules in methylene chloride was dissolved and then the mixture was diluted with 0.1N hydrochloric acid. After the evaporation of methylene chloride and the subsequent removal of EC, Au, and the stabilizing agent via filtration, the absorbance of the loaded cisplatin at 280 nm was measured with a UV–VIS spectrophotometer. The measured absorbance was then converted to the amount of cisplatin based on a standard calibration curve, which was previously constructed with the UV–VIS spectrophotometer on 0.1 N hydrochloric acids, each containing a known amount of cisplatin. The same procedure was repeated for iron oxide nanoparticles. The drug-loaded nanocapsules were tested for drug release in two types of media, 0.1 M HCl and phosphate buffer saline 0.1 M (pH = 7.4). Desorption profiles were obtained and the release of drugs were determined by UV-Vis spectra at a wavelength of 280 nm.

In addition, a statistical comparison was carried out by calculating average time, standard deviation and coefficient of variation for the drug release. 

$\mathrm{Mean}\left(\overset{\text{̅}}{x}\right)=\sum \frac{\mathrm{Dix}}{\mathrm{Di}}$

$\mathrm{Coefficient of Variation}=(\overset{\text{̅}}{X}/\sigma )\times 100$

## Results and Discussion

*UV-Vis spectrophotometer*

**Figure 3:**
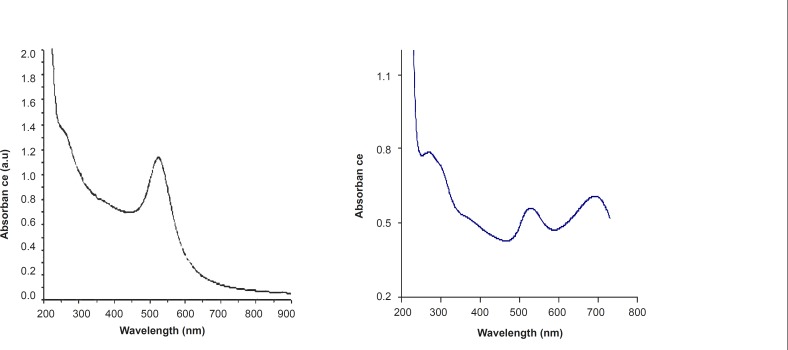
UV-Vis Spectrum of (a) Au and (b) Cis-Au

**Figure 4:**
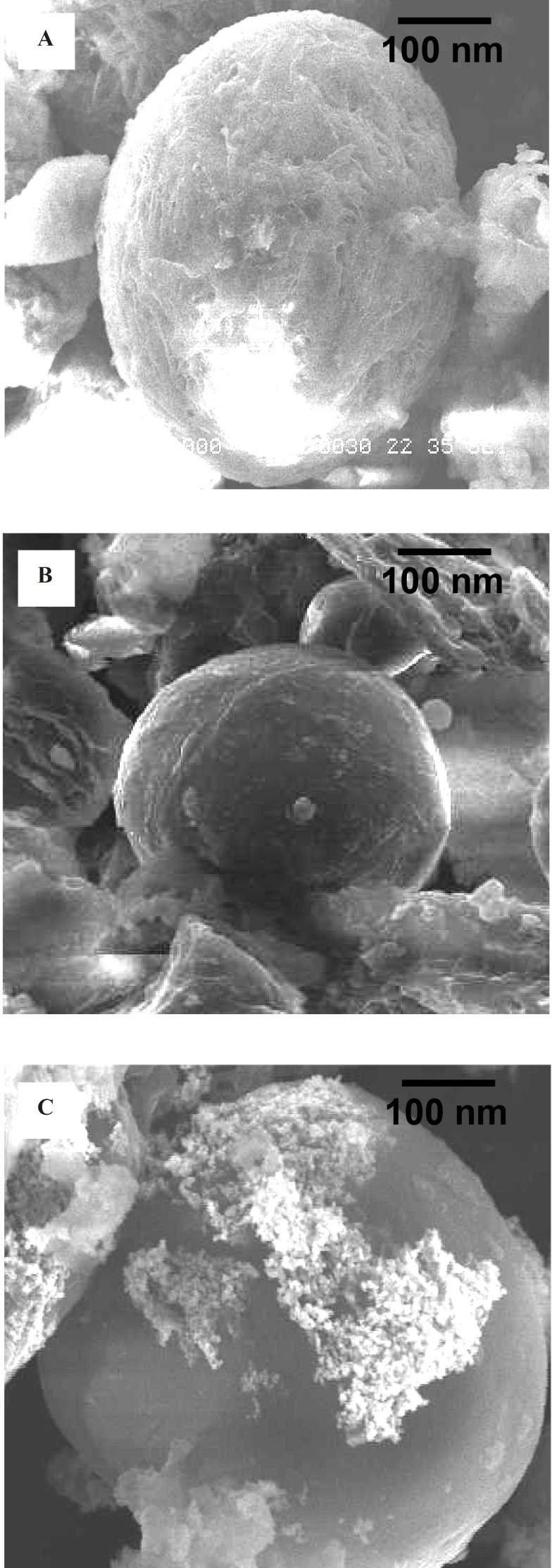
(a) SEM of EC/Cis; (b) SEM of EC/Cis-Au; (c) SEM of EC/Cis-Fe_3_O_4_

Figure 4a shows the UV-Vis spectrum of citrate stabilized gold nanoparticles. The plasmon band observed for the wine-red colloidal gold at 518 nm (Figure 3a) is the characteristic of gold nanoparticles. The pure drug shows a maximum at 280 nm and with the addition of Cisplatin to colloidal gold, both the bands at 280 and 518 nm pertaining to pure drug and Au colloids decrease in intensity steadily with time. This decrease is accompanied by emergence of an additional peak at 710 nm (Figure 3b); *i.e*. a change from wine-red to blue with the addition of drug to colloidal gold. 

The appearance of the new peak is due to the aggregation of gold nanoparticles and the replacement of citrate by cisplatin leading to the formation of gold–drug complex. Citrate ions are readily replaced by -NH ligand on gold nanoparticle surfaces. This ligand exchange reaction provides an important means for the chemical functionalization of the nanoparticles and greatly extends the versatility of these systems.

*Scanning electron microscope (SEM)*

Morphology of all the prepared nanocapsules (EC/Cis, EC/-Au and EC/Cis-Fe_3_O_4_) has been characterized by the SEM analysis (Figures 4a, 4b and 4c). EC/Cis-Au and EC/Cis nanocapsules were easily distinguished from EC/Cis nanocapsules by their color. While the color of EC/Cis microcapsule was white, EC/Cis-Au microcapsule was purple/blue because of the gold nanoparticles. 

The surface topography of EC/Cis-Au nanocapsules was smooth which was seen in SEM photographs. The SEM micrographs manifested that our nanocapsules had a nearly spherical shape.

*Transmission electron microscopy (TEM)*

**Figure 5:**
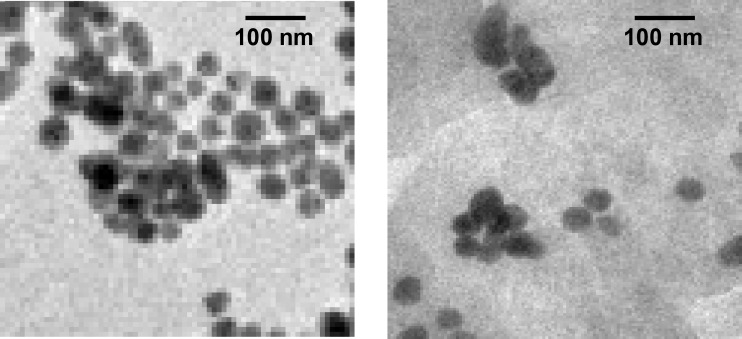
(a) TEM of EC/Cis-Au; (b) TEM of EC/Cis-Fe_3_O_4_.

Figures 5a and 5b show TEM images of ethylcellulose-coated Cis-Au nanoparticles. With the addition of drug to the gold nanoparticles, aggregation of gold nanoparticles takes place, which was observed in TEM images. Furthermore, the TEM image of EC/Cis-Au and EC/Cis-Fe_3_O_4 _confirms the presence of aggregated metal nanoparticles in the polymer nanocapsules. The average size of nanocapsules was found to be in the range of 100-300 nm.

**Table 1:** Drug release of cisplatin in 0.1 M HCl

**0.1 M HCl**			
**EC/Cis-Au**		**EC/Cis-Fe**_3_ **O**_4_	**EC/Cis**
**Time (h)** **(x)**	**% drug** **(D**_1_ **)**	**% drug** **(D**_2_ **)**	**% drug** **(D**_3_ **)**
0	4	8	17
1	6	10	35
2	8	16	51
4	15	30	62
8	24	45	77
12	32	53	86
24	46	60	93

**Table 2:** Drug release of cisplatin in 0.1 M PBS

**0.1 M PBS**			
**EC/Cis-Au**		**EC/Cis-Fe**_3_ **O**_4_	**EC/Cis**
**Time (h)** **(x)**	**%drug** **(D**_4_ **)**	**%drug** **(D**_5_ **)**	**%drug** **(D**_6_ **)**
0	13	17	22
1	18	24	49
2	32	40	65
4	50	54	75
8	57	62	85
12	62	66	90
24	69	75	98

*Infra-red characteristics (FT-IR)*

The characteristic band of magnetite is present at 610 cm^-1^ but not in the spectrum of magnetite free EC sub micron particles. In addition, the peaks around 3000 cm^-1^ are probably due to the contribution of CH_2_ stretch mode, both from oleate and EC molecules suggesting the presence of OA coated magnetite nanoparticles in the EC matrix.

**Figure 6:**
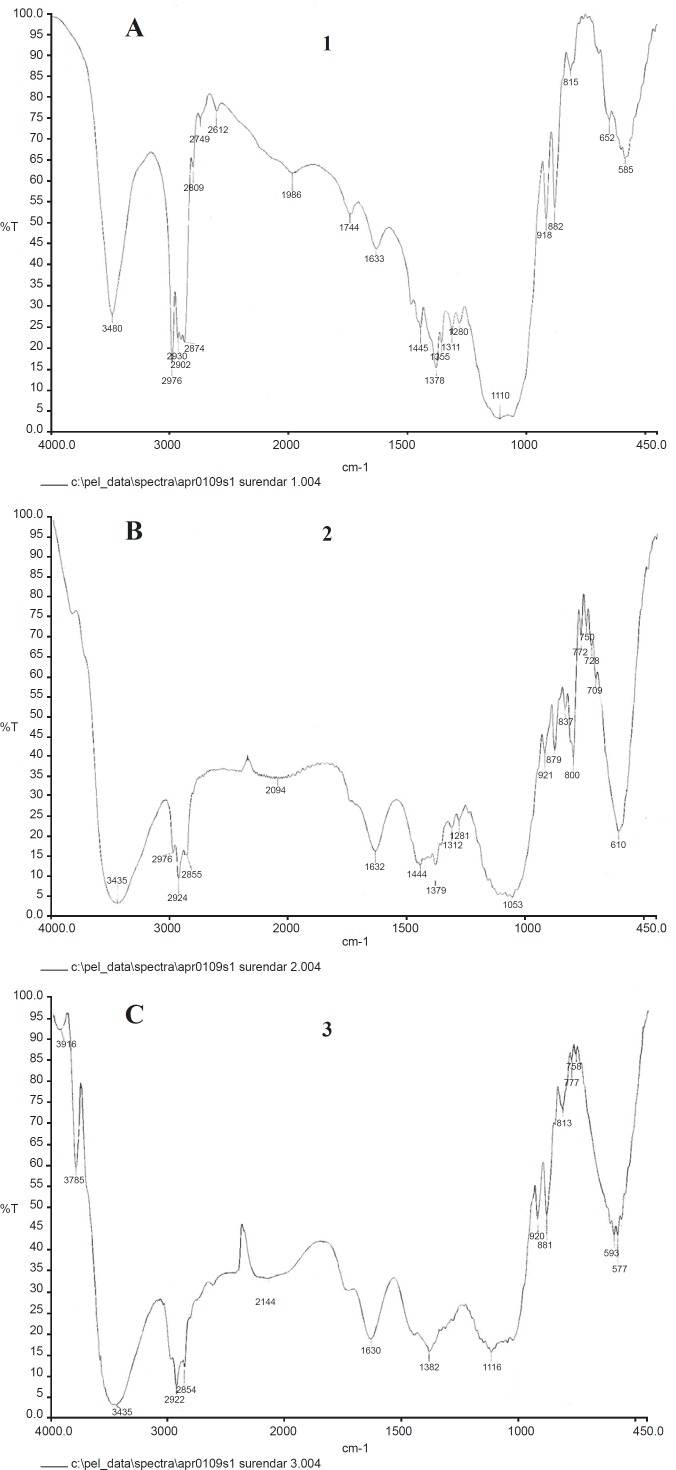
(a) FT-IR of EC/Cis-Au; (b) FT-IR of EC/Cis-Fe_3_O_4_; (c) FT-IR of EC/Cis

The peak values of gold incorporated nanocapsules are shifted to 3480 cm^-1^ for –NH_2_ due to the complex formation with cisplatin drug. The characteristic amine stretching peak of Cisplatin (3400-3200 cm^-1^), the asymmetric amine bending (1600-1500 cm^-1^) and the symmetric amine bending (1300-1200 cm^-1^) were observed in the Figures 6a, 6b and 6c.

*Drug release study*

**Figure 7:**
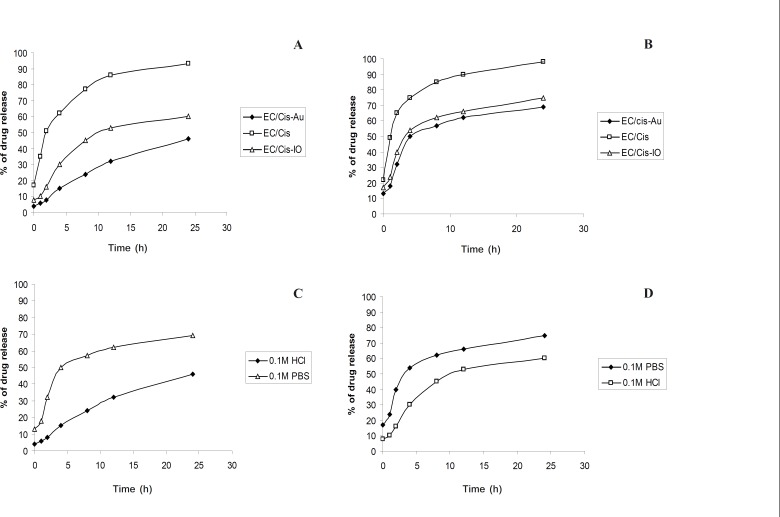
Drug release profiles of nanocapsules in (a) 0.1 M HCl (b) 0.1 M PBS. Drug release profiles using (c) gold nanoparticles (d) ironoxide nanoparticles

The release profiles of Cisplatin from EC/Cis, EC/Cis-Au and EC/Cis-Fe_3_O_4_ nanocapsules in the hydrochloric acid (0.1 M) and the phosphate buffered saline (pH 7.0) of 37 ± 0.1°C are shown in Figures 7a and 7b. Tables 1 and 2 show the drug release percentage of cisplatin in 0.1M HCl and 0.1 M PBS, respectively. Desorption profiles were obtained as follows. A mass of 0.03 g of drug encapsulated polymer nanoparticles were mixed with 5 mL of phosphate buffer solution in five fractions. Each fraction was centrifuged as a function of time. The absorbance of each solution was monitored at different times. Each sample solution was used just once so that there was no change in the concentration of the solution. The intensity of absorption was plotted against the time which gave the desorption profile of Cisplatin. Similar procedure is adopted for 0.1 M HCl solution.

From the Figures, it was understood that the drug release was slow and sustained in EC/ Cis-Au. The release rate of Cisplatin for EC/ Cis-Au nanocapsules was slower than that of EC/Cis-Fe_3_O_4 _and EC/Cis nanocapsules in both the dissolution media. This may be due to the smoother surface topography of EC/Cis-Au nanocapsules with smaller pores. 

EC/Cis-Au nanocapsules had slower release behavior mainly since the gold nanoparticle in nanocapsules hindered the diffusion of Cisplatin away from the nanocapsules. An initial burst effect (*i.e. *the rapid release of Cisplatin) of EC/ Cis nanocapsules was observed as shown in the Figures 7a and 7b. This initial rapid release could be attributed to those big pores on the surface of EC/Cis nanocapsules. 

The effect of gold nanoparticles on the release rate was particular importance because the release rates of Cisplatin for EC/Cis nanocapsules (93% in 0.1 M HCl and 98% in PBS), EC/Cis-Fe_3_O_4_ (60% in 0.1 M HCl and 75% PBS) were higher than the EC/Cis-Au nanocapsules (46% in 0.1 M HCl and 69% in PBS). This was a direct consequence of the smaller size of nanocapsule (formed under the higher shear stress) which produced a larger surface area leading to an increased release rate. 

Furthermore, From Figures 7c and 7d, the release rates of Cisplatin from EC/Cis, EC/ Cis-Au and EC/Cis-Fe_3_O_4_ nanocapsules were relatively slower in 0.1 M HCl than in pH 7.0 PBS (Figures 7c and 7d). This observation was attributed to the fact that the dissolution medium had a strong influence on the solubility drug and the solubility of Cisplatin in phosphate buffer saline was higher than in 0.1 M HCl at the same temperature. 

Tables 3 and 4 show the calculations of the mean, standard deviation and coefficient of variation for EC/cis, EC/cis-Au and EC/cis- Fe_3_O_4_ polymer nanocapsules. The results were summarized in Table 5. It was found that the coefficient of variation for nanocapsule with gold was less in both media which confirms that polymer nanocapsule with gold was consistent. From the mean time, the average amount of drug release for EC/cis-au was calculated based on Lagrange’s Interpolation Formula and was found to be 33.45% in 0.1 M HCl and 60.24% in 0.1 M PBS.

**Table 3:** Calculated values for mean, standard deviation and coefficient of variation for polymer nanocapsules in 0.1 M HCl

**X**	**D**_1_	**D**_1_ **X**	**D**_1_ **X** ^2^	**D**_2_	**D**_2_ **X**	**D**_2_ **X** ^2^	**D**_3_	**D**_3_ **X**	**D**_3_ **X** ^2^
0	4	0	0	8	0	0	17	0	0
1	6	6	6	10	10	10	35	35	35
2	8	16	32	16	32	64	51	102	204
4	15	60	240	30	120	480	62	248	992
8	24	192	1536	45	360	2880	77	616	4928
12	32	384	4608	53	636	7632	86	1032	12384
24	46	1104	26496	60	1440	34560	93	2232	53568
	ΣD_1_=135	Σ D_1_X= 1762	Σ D_1_X^2^ = 32918	Σ D_2_=222	ΣD_2_X=2598	Σ D_2_X^2^=45626	ΣD_3_=421	ΣD_3_X=4265	ΣD_3_X^2^=72111

**Table 4:** Calculated values for mean, standard deviation and coefficient of variation for polymer nanocapsules in 0.1 M PBS

**X**	**D**_4_	**D**_4_ **X**	**D**_4_ **X** ^2^	**D**_5_	**D**_5_ **X**	**D**_5_ **X** ^2^	**D**_6_	**D**_6_ **X**	**D**_6_ **X** ^2^
0	13	0	0	17	0	0	22	0	0
1	18	18	18	24	24	24	49	49	49
2	32	64	128	40	80	160	65	130	260
4	50	200	800	54	216	864	75	300	1200
8	57	456	3648	62	496	3968	85	680	5440
12	62	744	8928	66	792	9504	90	1080	12960
24	69	1656	39744	75	1800	43200	98	2352	56448
	ΣD_4_=301	Σ D_4_X=3138	Σ D_4_X^2^=53266	Σ D_5_=338	ΣD_5_X=3408	Σ D_5_X^2^=57720	ΣD_6_=484	ΣD_6_X=4591	ΣD_6_X^2^=76357

From the Figures, it was understood that the drug release was slow and sustained in EC/Cis-Au. The release rate of Cisplatin for EC/Cis-Au nanocapsules was slower than that of EC/Cis-Fe_3_O_4_ and EC/Cis nanocapsules in both the dissolution media. This may be due to the smoother surface topography of EC/Cis-Au nanocapsules with smaller pores. 

EC/Cis-Au nanocapsules had slower release behavior mainly since the gold nanoparticle in nanocapsules hindered the diffusion of Cisplatin away from the nanocapsules. An initial burst effect (*i.e. *the rapid release of Cisplatin) of EC/Cis nanocapsules was observed as shown in the Figures 7a and 7b. This initial rapid release could be attributed to those big pores on the surface of EC/Cis nanocapsules. 

The effect of gold nanoparticles on the release rate was particular importance because the release rates of Cisplatin for EC/Cis nanocapsules (93% in 0.1 M HCl and 98% in PBS), EC/Cis-Fe_3_O_4 _(60% in 0.1 M HCl and 75% PBS) were higher than the EC/Cis-Au nanocapsules (46% in 0.1 M HCl and 69% in PBS). This was a direct consequence of the smaller size of nanocapsule (formed under the higher shear stress) which produced a larger surface area leading to an increased release rate. 

Furthermore, From Figures 7c and 7d, the release rates of Cisplatin from EC/Cis, EC/Cis-Au and EC/Cis-Fe_3_O_4_ nanocapsules were relatively slower in 0.1 M HCl than in pH 7.0 PBS (Figures 7c and 7d). This observation was attributed to the fact that the dissolution medium had a strong influence on the solubility drug and the solubility of Cisplatin in phosphate buffer saline was higher than in 0.1 M HCl at the same temperature. 

Tables 3 and 4 show the calculations of the mean, standard deviation and coefficient of variation for EC/cis, EC/cis-Au and EC/cis-Fe_3_O_4_ polymer nanocapsules. The results were summarized in Table 5. It was found that the coefficient of variation for nanocapsule with gold was less in both media which confirms that polymer nanocapsule with gold was consistent. From the mean time, the average amount of drug release for EC/cis-au was calculated based on Lagrange>s Interpolation Formula and was found to be 33.45% in 0.1 M HCl and 60.24% in 0.1 M PBS.

**Table 5:** Comparison of statistical data for various nanocapsules

**S. No.**	**Nanocapsules**	**Mean**	**Standard deviation**	**Coefficient of variation**	**Average drug release (%)**
1	EC/cis-Au (0.1M HCl)	13.05	8.57	65.68	33.45
2	EC/cis-Fe_3_O_4_ (0.1M HCl)	11.70	8.28	70.76	52.59
3	EC/cis(0.1M HCl)	10.13	8.28	81.79	82.44
4	EC/cis-Au (0.1M PBS)	10.42	8.26	79.24	60.24
5	EC/cis-Fe_3_O_4_(0.1M PBS)	10.08	8.31	82.46	64.45
6	EC/cis (0.1M PBS)	9.48	8.23	86.80	87.31

## Conclusion

Colloidal gold and iron oxide nanoparticles were synthesized and the study of the encapsulation of Cisplatin to polymer nanoparticles was carried out using different analytical techniques. The aggregations of gold and iron oxide nanoparticles were ascertained using UV-Vis spectroscopy and TEM analysis. The morphology of Nanocapsules was studied using the scanning electron microscopy. In the present study, EC nanocapsules were prepared by solvent evaporation method for prolonged enteric release. The rate of drug release from the nanocapsules was influenced by the pH of the dissolution medium. FT-IR studies did not indicate any significant drug interactions. Due to the prolonged drug release in acidic pH as suggested by the dissolution, EC nanospheres can be used for delivery of drugs. Further, it was observed that the rate of drug release was slow and sustained in the case of EC/Cis-Au compared to that of EC/Cis-Fe_3_O_4_ nanocapsules and EC/Cis nanocapsules due to the better interaction between the drug and gold/iron oxide nanoparticles. It was also confirmed statistically that EC/cis-au nanocapsules were consistent and sustained. The combination of gold/iron oxide and polymer with Cisplatin results in an effective complex, where such complex can be used to release in a controlled rate as well as targeted places.
